# Comparison of three different anti-Xa assays in major orthopedic surgery patients treated with direct oral anticoagulant

**DOI:** 10.1186/s12959-017-0150-4

**Published:** 2017-10-12

**Authors:** Makoto Ikejiri, Hideo Wada, Shine Tone, Hiroki Wakabayashi, Masahiro Hasegawa, Takeshi Matsumoto, Naoki Fujimoto, Norikazu Yamada, Masaaki Ito, Kaname Nakatani, Akihiro Sudo

**Affiliations:** 1 Department of Central Laboratory, Mie University Graduate School of Medicine, Tsu, Japan; 2 Department of Molecular and Laboratory Medicine, Mie University Graduate School of Medicine, Tsu, Japan; 3 Department of Orthopaedic Surgery, Mie University Graduate School of Medicine, Tsu, Japan; 4 Department of Blood Transfusion Service, Mie University Graduate School of Medicine, Tsu, Japan; 5 Department of Cardiology and Nephrology, Mie University Graduate School of Medicine, Tsu, Japan; 6 Department of Laboratory Medicine, Mie University Graduate School of Medicine, 2-174 Edobashi, Tsu -City, Mie-ken 514-8507 Japan

**Keywords:** Deep vein thrombosis (DVT), Anti-Xa activity, DOAC, Prophylaxis, Orthopedic surgery

## Abstract

**Background:**

Measurement of edoxaban plasma concentration has been gathering attention in major orthopedic surgery patients receiving edoxaban for the prevention of venous thromboembolism (VTE).

**Methods:**

The anti-Xa activity was measured one hour after edoxaban intake using 3 different assays in 200 patients after major orthopedic surgery.

**Results:**

The anti-Xa activities on Day 8 were significantly higher than those on Day 4 and those on Day 4 were significantly higher than those on Day 1. The anti-Xa activities in two assays closely correlated with each other, but the other anti-Xa assay did not correlated with other two assays. The anti-Xa activities as detected in the three Xa assays were significantly higher in the patients without deep vein thrombosis (DVT) than in those with DVT on Day 4. Additionally, there were no significant differences in the anti-Xa activities of assays A, B and C between patients with and without massive bleeding (MB) on Days 1, 4, 8 and 15.

**Conclusion:**

The results of this study suggest that anti-Xa level could be predictive of the risk of VTE, but not of the risk of massive bleeding.

## Background

Major orthopedic surgery is associated with a high rate of postoperative venous thromboembolism (VTE) [1, 2]. The incidence of VTE, such as deep vein thrombosis (DVT) and pulmonary embolism (PE), is reported to range from 42% to 57% after total hip arthroplasty (THA) and 41% to 85% after total knee arthroplasty (TKA) [3] in the absence of thromboprophylaxis. VTE is one of the most prevalent cardiovascular diseases [4–6] and a major complication of major orthopedic or abdominal surgery. PE is a potentially fatal disease and is typically caused by proximal DVT. As patients with PE have non-specific and highly variable symptoms, and the imaging tests for a definitive diagnosis are expensive, the early diagnosis of PE is often difficult [7–9]. Therefore, preventing the development of VTE is clinically important after surgery.

The efficacy of low-molecular-weight heparins (LMWH) [10, 11] and fondaparinux [12, 13] for VTE prophylaxis has been established in major orthopedic surgery patients. However, there are a few cases of massive bleeding (MB) in patients receiving LMWH or fondaparinux [14–16]. Direct oral anticoagulants (DOACs) including both direct factor Xa inhibitors (rivaroxaban, apixaban and edoxaban) and the direct thrombin inhibitor (dabigatran) have shown a non-inferior effect for VTE prophylaxis compared to conventional prophylaxis [17].

The levels of LMWH, fondaparinux and DOACs cannot be monitored by routine assays such as activated partial thromboplastin time (APTT) or prothrombin time (PT); therefore, drug-specific anti-Xa assays use a chromogenic substrate to measure the concentration of anticoagulants that inhibit factor Xa in patients being treated with LMWH, fondaparinux or DOACs [18]. The anti-Xa activity results are reported to correlate with the weight and body mass index (BMI) and to predict MB in orthopedic patients with a normal renal function treated with fondaparinux [19].

We evaluated the anti-Xa activity measured using 3 different chromogenic anti-Xa assays (two assays available in Japan for the measurement of heparin and fondaparinux respectively, and one edoxaban-specific investigational assay) in 200 patients who underwent major orthopedic surgery and were treated with edoxaban for the prophylaxis of DVT in order to (i) examine the relationships between the results generated with these 3 assays and (ii) to investigate the potential clinical relevance of test results.

## Methods

### Patients

Patients presenting for THA and TKA at Mie University Hospital from January 1, 2014, to December 31, 2015 and receiving edoxaban 30 mg (Daiichi-Sankyo, Tokyo, Japan) once daily for VTE prophylaxis were enrolled in the study.

Screening for DVT was performed by a whole-leg compression ultrasound examination using the standardized ultrasound criteria for venous non-compressibility before the operation, as well as on Day 4 and 14 [20].

### Anti-Xa activity

Anti-Xa activity was measured prospectively on Days 1, 4, 8 and 15. Blood was drawn one hour after drug intake on Days 1, 4 and 8 and 12 h after the last drug intake on Day 15.

The anti-Xa activity of edoxaban was measured using (i) Testzym^Ⓡ^Heparin S (Sekisui Medical Co., Ltd., Tokyo, Japan) on the Coagrex^Ⓡ^ 800 System (Sekisui; assay A) [15, 19]; (ii) STA®-Liquid Anti-Xa (Stago, Asnières-sur-Sreine, France) on STA^Ⓡ^-R Evolution® coagulometer (Stago; assay B) with a dedicated test set-up [21]; and (iii) HemosIL® Liquid Heparin (Instrumentation Laboratory; Bedford, MA, USA) on ACL-TOP® (Instrumentation Laboratory; assay C) [21, 22].

Assay A was calibrated using a fondaparinux-specific standard and results were expressed in fondaparinux mg/L. Assay B was calibrated using a edoxaban-specific calibrator set (STA®Edoxaban calibrator) and verified using an edoxaban-specific control set (STA®Edoxaban Control), both developed by Stago. Assay C was calibrated using the HemosIL Liquid Heparin Calibrator (Instrumentation Laboratory). Results for assay C were expressed in heparin international units (IU)/mL. Assay A contains additional antithrombin (AT) in the assay system, while assays B and C do not.

### Statistical analysis

The data are expressed as the medians and 25th–75th percentiles. The differences between the groups were examined using the Mann-Whitney U-test. The analysis among 4 groups was performed by the one-factor analysis of variance (ANOVA). The correlation was analysed by Spearman’s rank correlation coefficient. A *p*-value <0.05 was considered to be statistically significant. All statistical analyses were performed using the Stat Flex, version 6, software package (Artec Co. Ltd., Osaka, Japan).

## Results

Two hundred orthopedic patients, including 134 THA and 66 TKA cases, treated with edoxaban and intermittent pneumatic compression for DVT prophylaxis from January 1, 2014, to December 31, 2015, were enrolled in this study (Table 1). These patients received 30 mg of edoxaban by oral administration once a day for 14 days beginning 24 h after the discontinuation of lumbar anesthesia. Of these 200 patients, 52 exhibited DVT, and 21 patients had MB, defined as a reduction in the hemoglobin level by >2 g/dl compared with that at Day 1 or a hemoglobin level < 7 g/dl [15]. As the registration for this trial was late in 26 patients, the Day 1 samples were not obtained in these patients. The sampling was also stopped after complication with DVT or MB.

**Table 1:** Anti-Xa activities in orthopedic patients with or without massive bleeding

	Anti-Xa activity					
	A (mg/L)		B (mg/L)		C (IU/mL)	
	With MB	Without MB	With MB	Without MB	With MB	Without MB
Day one	0.08 (0.03–0.14)	0.08 (0.01–0.22)	0.05(0.00–0.13)	0.08 (0.01–0.15)	0.29 (0.01–0.54)	0.32 (0.03–0.81)
Day four	0.21 (0.09–0.28)	0.20 (0.06–0.31)	0.14 (0.07–0.18)	0.13 (0.04–0.23)	0.61 (0.25–1.01)	0.69 (0.15–1.30)
Day eight	0.13 (0.05–0.25)	0.24 (0.12–0.37)	0.11 (0.02–0.20)	0.16 (0.09–0.26)	0.70 (0.13–1.28)	0.81 (0.25–1.41)
Day fifteen	0.02 (0.00–0.04)	0.03 (0.01–0.06)	0.01 (0.01–0.02)	0.02 (0.01–0.03)	0.03 (0.00–0.09)	0.08 (0.04–0.13)

There were no significant differences in the anti-Xa activities according to the assay used between the patients with and without massive bleeding

*MB* massive bleeding

**Fig. 1:**
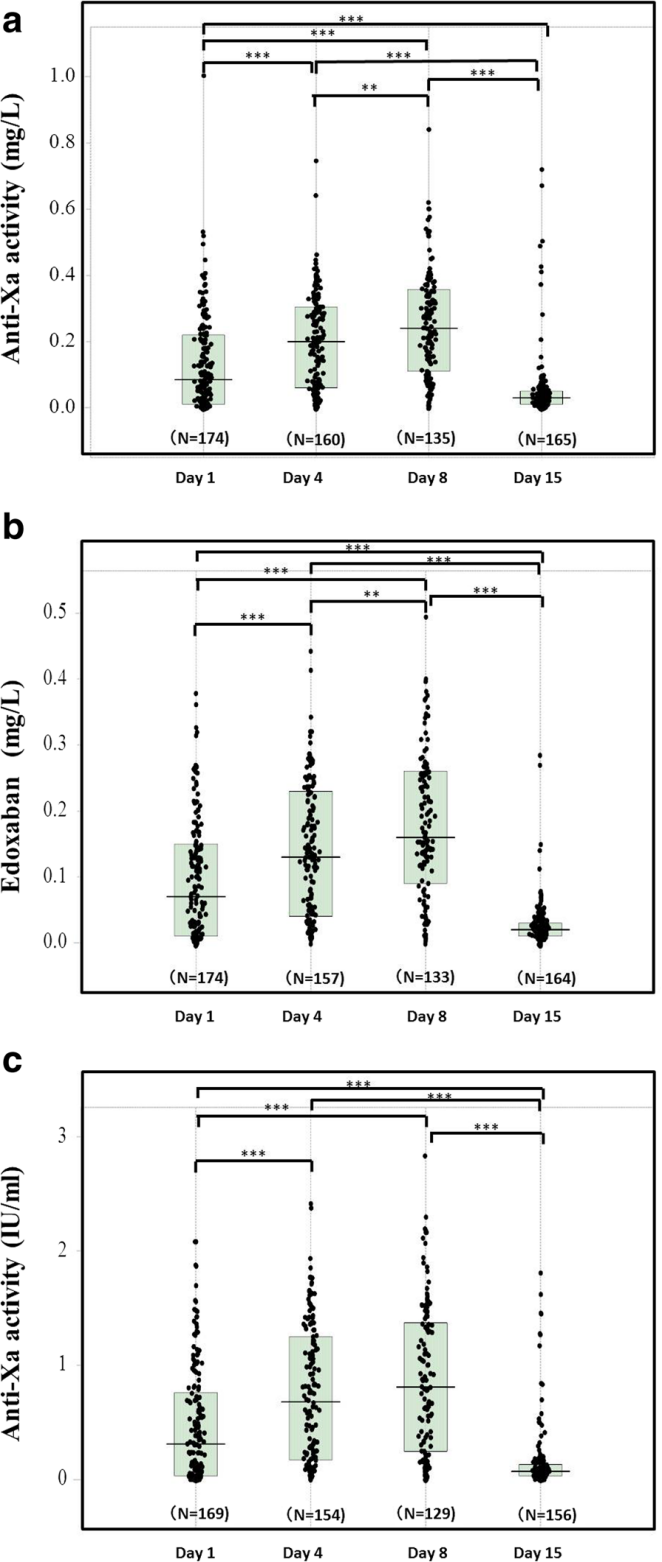
The anti-Xa activity (A), (B) and (C) in orthopedic patients treated with edoxaban during TKA or THA. The anti-Xa activities of A kit (**a**), B kit (**b**) and C kit (**c**) in orthopedic patients treated with edoxaban on Days 1, 4, 8 and 15. ***, *p* < 0.001; **, *p* < 0.01; *, *p* < 0.05

**Fig. 2:**
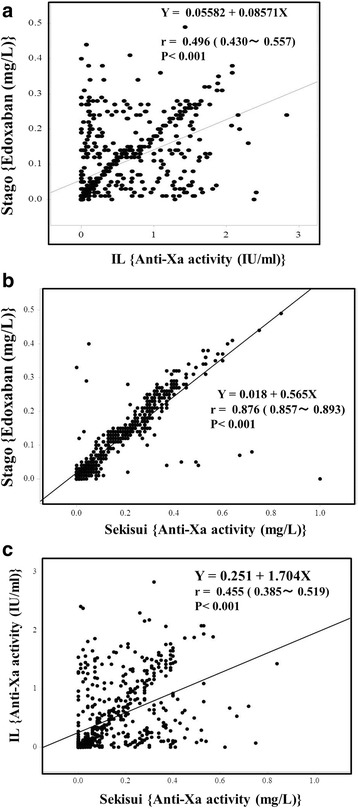
Correlation between the anti-Xa activities of A and B assay (**a**), A and C assay (**b**) and B and C assay (**c**)

The anti-Xa activities on Day 8 (assay A [*p* < 0.01], 0.24 mg/L [0.11–0.36 mg/L]; assay B [p < 0.01], 0.16 mg/L [0.09–0.26 mg/L]; assay C [not significant], 0.81 IU/mL [0.25–1.37 IU/mL]) were higher than those on Day 4 (assay A, 0.20 mg/L [0.06–0.31 mg/L]; assay B, 0.13 mg/L [0.04–0.23 mg/L]; assay C, 0.68 IU/mL [0.17–1.25 IU/mL] and the those levels (on Day 4) were significantly higher than those on Day 1 (assay A, 0.08 mg/L [0.01–0.21 mg/L]; assay B, 0.07 mg/L [0.01–0.15 mg/L]; assay C, 0.31 IU/mL [0.03–0.76 IU/mL]) (*p* < 0.001 for each) (Fig. 1a–c). ANOVA showed that the variation between subgroup was significant (p < 0.001, respectively) in three assays. The anti-Xa activities in assay A closely correlated with those in assay B (Y = 0.018 + 0.565X, *r* = 0.876 and p < 0.001), but the correlations between assays A and C (0.251 + 1.704X, *r* = 0.455 and *p* < 0.001) and assays B and C (0.056 + 0.086X, *r* = 0.496 and p < 0.001) were not close (Fig. 2a, b, and c).

**Fig. 3:**
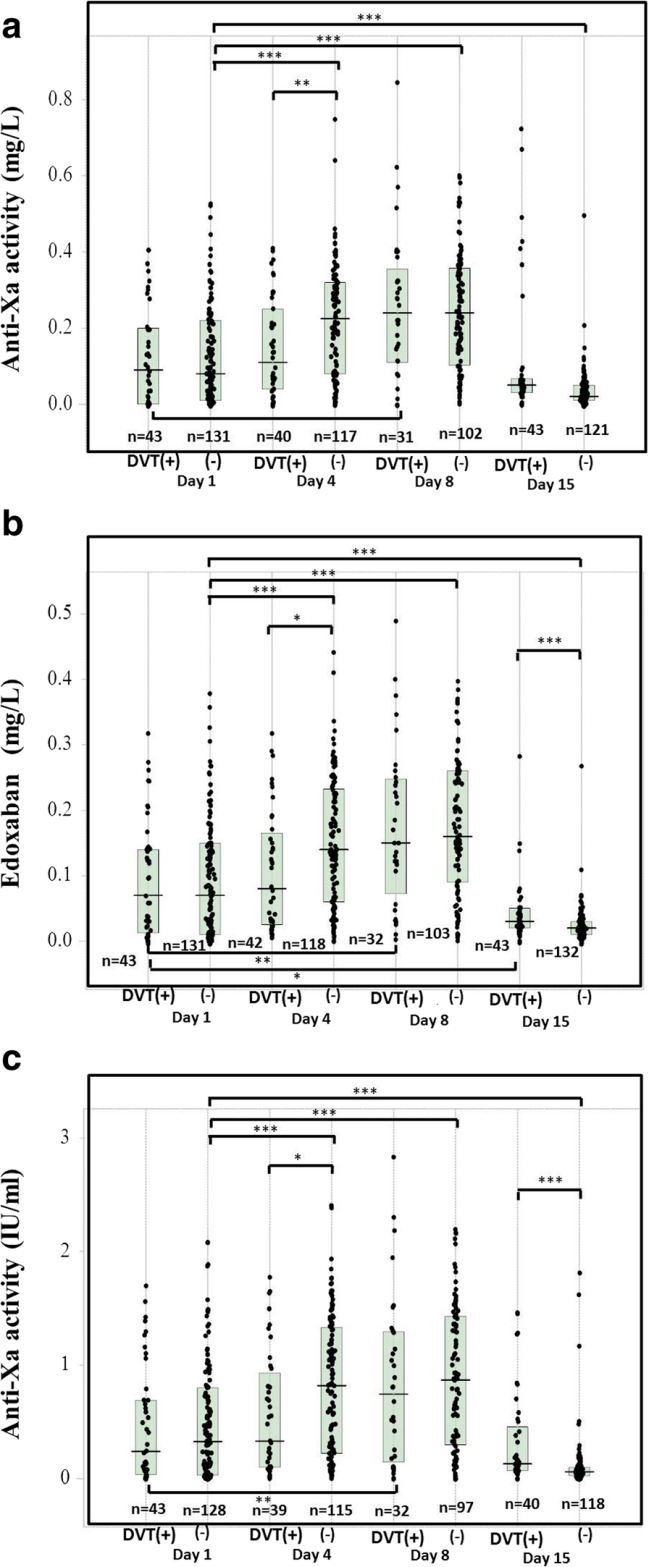
The anti-Xa activity in orthopedic patients with and without DVT treated with edoxaban during TKA or THA. The anti-Xa activities of A kit (**a**), B kit (**b**) and C kit (**c**) in orthopedic patients treated with edoxaban on Day 1, 4, 8 and 15. ***, p < 0.001; **, p < 0.01; *, p < 0.05

The anti-Xa activities were significantly higher in the patients without DVT than in those with DVT on Day 4 (assay A: *p* < 0.01, assays B and C: *p* < 0.05) and the anti-Xa activities of assay B and C (p < 0.001 for both) were significantly higher in the patients with DVT than in those without DVT on Day 15 (Fig. 3a–c). ANOVA showed that the variation between subgroup was significant (p < 0.001, respectively) in three assays. The antiXa activity in more than half of DVT patients was significantly low on Day 4. Although there were no significant differences in the anti-Xa activities between on Days 1 and 4 in the patients with DVT, the anti-Xa activities of assays A, B and C in patients without DVT were significantly higher (p < 0.001, respectively) on Day 4 than on Day 1 (p < 0.001). In addition, there were no significant differences in the anti-Xa activities of assays A, B and C between patients with and without MB on Day 1, 4, 8 and 15 (Table 1).

## Discussion

Global coagulation tests such as APTT and PT are not recommended for selective anti-Xa drug plasma level evaluation due to inconsistent sensitivity across drugs and reagents and a lack of specificity with these assays; an anti-Xa assay is required to monitor these drugs [18]. However several issues have been encountered with anti-Xa assays, such as their complicated nature, cost and a little evidences supporting their clinical use, except in emergency situations (e.g. major bleeding, surgery or invasive procedure). Although three different anti-Xa assays are available, there have been few reports comparing the results of these assays [23]. In a previous report [23], the activities detected with these assays were well correlated in patients treated with fondaparinux. In the present study, the anti-Xa activities in assay A closely correlated with those in assay B, but the correlations were poor between assays A and C and assays B and C. Anti-Xa assay A contains additional AT in the assay system, while anti-Xa assays B and C do not, suggesting that the existence of AT was not the cause for the poor correlation in the anti-Xa activity. Instead, the poor correlation may have been due to the fact that DOAC is a direct anticoagulant for Xa without activation of AT.

There are nevertheless limitations to the comparison of the three assays used in this study. In fact, one has to keep in mind that assays should preferably use for the indication that they have designed for. Even though heparin, fondaparinux and edoxaban exhibit anti-Xa activity, they differ in many ways [24].

The distribution of anti-Xa activity ranged more widely in the patients treated with edoxaban than in those treated with fondaparinux [15, 23]. This wide distribution in the anti-Xa activity of edoxaban may be the cause of the characteristic pharmacokinetics and pharmacodynamics of edoxaban [25]. The anti-Xa activity gradually increased from Day 1 to 8, suggesting that the anti-Xa activity may accumulate, necessitating anti-Xa monitoring in patients being treated with edoxaban. As an anti-Xa activity was not high in the patients treated with edoxaban comparison with fondaparinux, low frequency of MB was observed in this study.

The anti-Xa activities were significantly lower in the patients with DVT than in those without DVT on Day 4, suggesting that lower anti-Xa activity may be a one of the causes for DVT in patients being treated with edoxaban. However, there were no significant differences in the anti-Xa activities detected by the assays between patients with and without MB, suggesting that anti-Xa activities may not be useful for predicting MB in these patients. These findings differed between fondaparinux and edoxaban treatments. Several reports [15, 19, 26] have shown that there were no significant differences in the anti-Xa activities between patients with and without DVT receiving fondaparinux treatment following major orthopedic surgery. These differences may be due to the differences in the mechanism for anticoagulation of these drugs; fondaparinux activates AT to inhibit Xa, while edoxaban directly inhibits Xa. Although the treatment of VTE requires high anti-Xa activity [27], VTE prophylaxis after orthopedic surgery may not require high anti-Xa activity [25]. Furthermore, the frequency of MB after orthopedic surgery in patients treated with edoxaban in this study may have been low compared with previous studies in patients treated with fondaparinux [15, 26].

## Conclusion

Although the three anti-Xa assays were not closely correlated with each other, these activities were significantly lower in the patients with DVT than in those without. There were no significant differences among the three assays regarding the usefulness of monitoring edoxaban treatment in orthopedic patients.

## Abbreviations

APTT: Activated partial thromboplastin time
AT: Antithrombin
BMI: Body mass index
DOACs: Direct oral anticoagulants
DVT: Deep vein thrombosis
LMWH: Low-molecular-weight heparins
MB: Massive bleeding
PE: Pulmonary embolism
PT: Prothrombin time
THA: Total hip arthroplasty
TKA: Total knee arthroplasty
VTE: Venous thromboembolism
